# Overnutrition, Hyperinsulinemia and Ectopic Fat: It Is Time for A Paradigm Shift in the Management of Type 2 Diabetes

**DOI:** 10.3390/ijms25105488

**Published:** 2024-05-17

**Authors:** Joseph A. M. J. L. Janssen

**Affiliations:** Department of Internal Medicine, Erasmus Medical Center, 3015 GD Rotterdam, The Netherlands; j.a.m.j.l.janssen@erasmusmc.nl, Tel.: +31-0612752413

**Keywords:** hyperinsulinemia, overnutrition, ultra-processed foods, insulin resistance, hepatic insulin clearance and insulin resistance, prediabetes, type 2 diabetes, bariatric surgery, VLCD diets, caloric restriction, prevention, personal fat hypothesis, ectopic fat, remission of type 2 diabetes, tirzepatide

## Abstract

The worldwide incidence of prediabetes/type 2 has continued to rise the last 40 years. In the same period, the mean daily energy intake has increased, and the quality of food has significantly changed. The chronic exposure of pancreatic β-cells to calorie excess (excessive energy intake) and food additives may increase pancreatic insulin secretion, decrease insulin pulses and/or reduce hepatic insulin clearance, thereby causing chronic hyperinsulinemia and peripheral insulin resistance. Chronic calorie excess and hyperinsulinemia may promote lipogenesis, inhibit lipolysis and increase lipid storage in adipocytes. In addition, calorie excess and hyperinsulinemia can induce insulin resistance and contribute to progressive and excessive ectopic fat accumulation in the liver and pancreas by the conversion of excess calories into fat. The personal fat threshold hypothesis proposes that in susceptible individuals, excessive ectopic fat accumulation may eventually lead to hepatic insulin receptor resistance, the loss of pancreatic insulin secretion, hyperglycemia and the development of frank type 2 diabetes. Thus, type 2 diabetes seems (partly) to be caused by hyperinsulinemia-induced excess ectopic fat accumulation in the liver and pancreas. Increasing evidence further shows that interventions (hypocaloric diet and/or bariatric surgery), which remove ectopic fat in the liver and pancreas by introducing a negative energy balance, can normalize insulin secretion and glucose tolerance and induce the sustained biochemical remission of type 2 diabetes. This pathophysiological insight may have major implications and may cause a paradigm shift in the management of type 2 diabetes: avoiding/reducing ectopic fat accumulation in the liver and pancreas may both be essential to prevent and cure type 2 diabetes.

## 1. Introduction

In the past 40 years, the incidence of type 2 diabetes has rapidly risen globally, (partly) driven by population growth, aging and increasing age-specific prevalence [1]. In 2008, the age-standardized adult diabetes prevalence was 9.8% (8.6–11.2) in men and 9.2% (8.0–10.5) in women, as compared to 8.3% (6.5–10.4) and 7.5% (5.8–9.6) in 1980 [1]. The number of people with diabetes increased from 153 (127–182) million in 1980 to 347 (314–382) million in 2008 [1]. In 2021, 537 million adults (20–79 years) were living with diabetes, and this number is predicted to rise to 643 million by 2030 and to 783 million by 2045 [2]. In addition, over three in four adults with diabetes lived in 2021 in low- and middle-income countries [2]. Recent data found that 14.3% of US adults (over 20 years of age) were diagnosed with diabetes, while 38% had developed prediabetes [3]. Thus, currently more than 50% of US adults have developed prediabetes or diabetes.

The increase in the prevalence of prediabetes/type 2 diabetes in the last 40 years may be related to a combination of an increased food intake and lifestyle changes resulting in lower calorie expenditure. The mean daily energy intake per person and the use of ultra-processed foods significantly increased in many countries in the world. For example, the average American consumed 2481 calories a day in 2010, which is about 23% more than in 1970 (total 2025 calories a day) [4]. This excess energy intake may have led to an increase in subcutaneous and ectopic fat mass [5]. The typical “modern” diet has a high content of sugars and high amounts of caloric sweeteners, corn-derived fructose syrup, saturated fats and proteins but a relatively lower content of fruits and vegetables (7.9% of the daily calorie intake in 2010 vs. 9.2% in 1970) [6]. In 2010, almost half of the daily calorie intake (46.6%) in the diet came from just two food groups: flours and grains (581 calories, or 23.4%) and fats and oils (575, or 23.2%) [4]. This is a remarkable increase from the combined 37.3% in 1970 [4]. A large part of the day, pancreatic β-cells are exposed to a complex milieu of nutrients. The β-cells function as “fuel sensors” and respond to dietary nutrients [2]. Although glucose is the primary stimulus for insulin secretion, (excess) lipids and amino acids can also increase insulin secretion even when circulating glucose levels are normal [7]. Insulin is the main anabolic hormone and transports glucose to insulin-dependent cells/tissues, such as the liver, muscle and adipose tissue, to maintain glucose homeostasis [8]. Insulin also enables the fat cell to take up and store fat as well following excessive energy intake [9]. In addition to its peripheral effects on glucose, lipid and protein metabolism, insulin is believed also to act in the brain to regulate normal energy balance [10]. The chronic and continuous exposure (day in, day out) of pancreatic β-cells to large amounts of food (as a result of excessive energy intake) may induce insulin hypersecretion. Varying amounts and durations of overfeeding can lead to elevated insulin levels in normoglycemic normal weight subjects suggesting that increased plasma insulin concentrations are a general feature of overfeeding [11,12,13]. This has important clinical significance, since many prospective longitudinal studies have demonstrated that in subjects with normal glucose tolerance, baseline hyperinsulinemia is a significant predictor of the development of prediabetes and type 2 diabetes during follow-up [14,15,16,17,18,19,20,21] (Table 1). In addition, increasing evidence suggests that fasting hyperinsulinemia itself may have a primary pathogenetic role in the development of insulin resistance and diabetes [17] (see below). Type 2 diabetes was seen for years as being a condition with an irreversible natural course: worsening over time was considered to be a consequence of progressive β-cell failure. However, increasing evidence shows that the remission of type 2 diabetes can be achieved through bariatric surgery and intensive lifestyle management [22]. Professor Roy Taylor postulated that type 2 diabetes is caused mainly by excess, yet reversible, (ectopic) fat accumulation in the liver and pancreas [23]. This new insight into the pathophysiology of type 2 diabetes may cause a paradigm shift in the management of type 2 diabetes: avoiding/reducing ectopic fat accumulation in the liver and pancreas may both be essential to prevent and cure type 2 diabetes (see below for more details).

## 2. Pancreatic Insulin Secretion Is Pulsatile in Healthy Subjects

As is the case for many other hormones, insulin is normally released by the pancreas in a rapid pulsatile manner leading to oscillatory insulin concentrations in the blood [24]. The pulsatile release of insulin into the portal vein generates 3–5 min oscillations, and the amplitude of insulin is markedly suppressed after passage through the liver [24,25]. In addition to the rapid pulsatile insulin release pattern, a slower ultradian oscillatory insulin pattern of 1–3 h has been described [26,27]. In healthy subjects, at least 75% of the insulin secretion is released in a pulsatile manner [26]. Pulsatile insulin may act more efficiently on insulin target tissues [28]. A major advantage of pulsatile insulin release is the prevention of the downregulation of the insulin receptors [24]. In contrast, exposing insulin receptors to continuous high insulin levels might downregulate and internalize the insulin receptor, leading to reduced insulin-mediated actions [29,30]. In support of this, numerous studies have reported that the cyclic variation in insulin keeps insulin receptors more sensitive than continuous insulin exposure [31,32,33]. Pulsatile insulin secretion may be also necessary for normal pancreatic β-cell function: recent data provide evidence for the positive role of insulin on insulin secretion and β-cell survival [34]. Pulsatile insulin secretion from pancreatic β-cells is altered during the progression from prediabetes to type 2 diabetes and contributes significantly to the pathogenesis of insulin resistance in a variety of circumstances [28,35,36,37]. Individuals prone to developing diabetes or with overt type 2 diabetes show irregular oscillations of plasma insulin [25,26]. The loss of the first-phase insulin response to intravenous glucose is one of the earliest detectable defects of β-cell dysfunction in prediabetes and type 2 diabetes mellitus [38]. Pulsatile insulin release leads to significant changes in plasma insulin levels in a short period of time. This renders the assessment of insulin secretion by only a single blood sample not very informative. When reliable plasma insulin levels are required, it is advised to calculate average plasma insulin concentrations from at least three blood samples drawn at approximately 5 min apart, during 15 min [31]; however, in common clinical practice and in many epidemiological studies, the amount of insulin is mostly measured in only one blood sample.

## 3. Long-Term Effects of Overfeeding during Critical Developmental Periods of Life and Hyperinsulinemia

During pregnancy, maternal physiology drastically changes to support fetal growth and development [39]. Mammals are exposed to two environments during their development: the in utero environment and the postnatal environment [40]. Metabolic imprinting/programming refers to long-term effects on the physiologic and metabolic responses of the offspring induced by an offspring’s prenatal and postnatal environment (in utero and early in postnatal life) [40]. Overnutrition during fetal and early life may have life-long deleterious effects on health later in life [41]. In gestational diabetes mellitus (GDM), the maternal β-cells can no longer adequately adapt to the increased food supply and insulin demands during late pregnancy. As a result, the blood glucose levels in the mother rise, which then causes increased insulin secretion (as an adaptive response) by the fetal pancreas. Thus, GDM pregnancies are accompanied by maternal hyperglycemia and fetal hyperinsulinemia, which both promote fetal overnutrition. When studying the consequences of early postnatal overnutrition in rats 1 yr. after birth, overfat rats still exhibited enhanced insulin secretion and an elevated content of GLUT-2 in pancreatic islets compared to the control group [41]. Thus, early postnatal overnutrition may have long-lasting effects on insulin secretion [42]. Epigenetic modifications in response to overnutrition may lead to permanent alterations (imprinting) of genes involved in glucose-stimulated insulin secretion [41]. Imprinting caused by a diabetic intrauterine environment can be transmitted across generations, and it has been suggested that this (partly) may explain the observed increases in hyperinsulinemia, prediabetes and type 2 diabetes seen in many countries over the past several decades [42,43].

## 4. Plasma Insulin Levels after Long-Term Overfeeding in Identical Twins

In response to long-term overfeeding with a mixed diet (4.2 MJ/d during a 100-day period), fasting insulin levels were found significantly increased in normal weight identical twins [11]. In addition, fasting plasma glucose levels were (slightly but) significantly increased by overfeeding. However, during a 75 g oral glucose tolerance test (OGTT), no major alterations in glucose tolerance were observed after these 100 days, although the insulin area under the curve during the OGTT and a mixed meal test was significantly increased [11]. The change in the insulin area under the curve during the OGTT after 100 days of overfeeding was positively correlated to changes in total subcutaneous fat, suggesting that changes in total subcutaneous fat represent an important correlate of insulin changes with overfeeding [11]. Moreover, during overfeeding, more fat was gained in the subcutaneous fat depots than in the visceral depots [11]. In response to overfeeding, high twin intrapair similarity was observed for the changes in fasting insulin levels [11]. However, changes in fasting plasma insulin concentration after overfeeding showed approximately six times more variance between identical twin pairs than within identical twin pairs, suggesting that some twins were more prone than others to modify their insulin levels after overfeeding [11]. Overall, these results suggest that chronic hypercaloric intake (overnutrition) increases pancreatic insulin secretion, leads to chronic hyperinsulinemia and increases subcutaneous fat [11]. In addition, it suggests that the genotype of an individual may be an important determinant of insulin responses during a prolonged positive energy balance period [11]. Insulin secretion appears to be more heritable than insulin resistance: in genome-wide association studies (GWASs), only a few loci were associated with insulin resistance and type 2 diabetes, while the majority of the loci identified by GWASs were associated with defects of the β-cell of the pancreas [44,45,46]. The rapid changes observed in the global epidemiology of type 2 diabetes are likely caused by environmental and behavioral factors overlaid on a background of genetic predisposition [47].

## 5. Changes in Food and Food Additives Play A Role in Calorie Intake and Hyperinsulinemia

Since the 1980s, the use of ultra-processed foods has become increasingly dominant worldwide [48]. The consumption of ultra-processed foods represents nowadays 50–60% of the daily energy intake in some high-income countries, and middle-income and low-income countries are following suit [49]. In 2009–2010, the average US daily energy intake was 8.6 MJ, with 5 MJ (57.6%) coming from ultra-processed foods [50]. Ultra-processed foods may facilitate overeating. They are typically high in calories, salt, sugar and fat and have been suggested to be engineered to have supernormal appetitive properties [51,52]. When subjects were randomly assigned to either an ultra-processed or unprocessed diet for 14 days, followed immediately by the alternate diet for the final 14 days, the ultra-processed diet caused increased ad libitum energy intake and weight gain, despite being matched to the unprocessed diet for presented calories, sugar, fat, sodium, fiber and macronutrients [53]. However, although it was attempted to match several nutritional parameters between the two diets, ultra-processed versus unprocessed meals differed substantially in the proportion of added total sugar (~54% vs. 1%, respectively), insoluble total fibers (~16% vs. 77%, respectively) and saturated total fat (~34% vs. 19%) [53]. In addition, it was found that ad libitum intake was ~500 kcal/day more in the ultra-processed vs. unprocessed diet [53]. The increased energy intake during the ultra-processed diet resulted from a greater intake of carbohydrates (280 ± 54 kcal/day) and fat (230 ± 53 kcal/day) but not of protein [53]. Thus, subjects ate more calories when exposed to a diet composed of ultra-processed foods compared to a diet composed of unprocessed foods. Moreover, the intake of ultra-processed food dose-dependently appeared to increase the risk of type 2 diabetes [54]. In 2011, professor Barbara Corkey hypothesized that that excessive β-cell secretory insulin responses, possibly to environmental factors, may be a major cause of type 2 diabetes [55]. She suggested that (ultra)processed food, some food additives and environmental toxins, which have entered our food supply the last four decades, are the main causes of hyperinsulinemia [56]. In favor of this hypothesis are several in vitro studies: the in vitro chronic exposure (during 24–72 h.) of pancreatic β-cells to elevated glucose and fatty acids induced basal insulin hypersecretion and impaired glucose-stimulated insulin secretion (GSIS) [57]. In addition, the in vitro administration of the glyceride mono-oleoyl-glycerol (MOG) stimulated basal insulin secretion from pancreatic ß-cells in a concentration-dependent manner without increasing intracellular Ca^2+^ or O_2_ consumption [57,58]. Artificial sweeteners such as sucralose, aspartame and saccharin, which are frequently present in modern foods, were shown to increase basal insulin secretion in rat islets [55,58]. Of further note, the amount of iron consumption in the diet has increased the last four decades as the consumption of the lean content of food animals has increased in many societies [55]. Interestingly, iron can also increase pancreatic insulin secretion without increasing intracellular Ca^2+^ [55,58]. MOG, artificial sweeteners and iron all stimulate pancreatic insulin secretion in the absence of glucose or another stimulatory fuel. It has been further suggested that all these agents and the excessive supply of fuels stimulate basal insulin secretion through an increased production (or less use) of NADH and also through more production (or less scavenging) of reactive oxygen species (ROS) [55,57]. Fructose occurs naturally in fruits and vegetables but is also increasingly added to many processed foods as table sugar (sucrose) and high-fructose corn syrup [59]. Data collected from 1998 to 1994 in the third National Health and Examination Survey (NHANES) showed that the mean fructose consumption in the USA had increased to 54.7 g/day (10.2% of total caloric intake), compared to 37 g/day (8%) of total intake in 1977–1978 [59]. It has been further suggested that fructose can exert a potentiating role on glucose-mediated insulin secretion [60]: chronic fructose exposure rendered rodent and human pancreatic β-cells hyper-responsive to intermediate glucose concentrations suggesting that fructose potentiates glucose-induced insulin secretion (GSIS) [61]. In addition, fructose consumption may induce a rise in glucagon-like peptide-1 (GLP-1) and so mediate hyperinsulinemia [62]. In line with these findings, an oral solution of sucrose (which is a disaccharide formed by glucose and fructose) potentiated insulin secretion compared to equimolar glucose alone [63]. The consumption during 5 weeks of a diet containing 15% of the calories as fructose resulted in significantly higher insulin and glucose responses than the consumption of diets containing 0% or 7.5% of the calories as fructose, suggesting that even moderate levels of dietary fructose can increase insulin secretion [64]. Thus, changes in the quality of food and the excessive consumption of food and drinks, containing large amounts of sugars/sweeteners/iron, may lead to hyperinsulinemia and excessive pancreatic insulin secretion. As a direct result of overeating, with frequent snacking and the consumption of sucrose-containing soft drinks during many hours of the day, plasma insulin levels are elevated for much of the day [65]. In addition, a high intake of simple sugars has been found to be related to an increased liver fat content and to nonalcoholic fatty liver disease. These liver changes may reduce hepatic insulin clearance and increase peripheral insulin levels (see further below paragraphs **Hepatic Insulin clearance and hyperinsulinemia** and **The personal fat threshold hypothesis and the development of type 2 diabetes** for more details) [66].

## 6. Hyperinsulinemia Precedes Decreased Insulin Receptor Sensitivity (Insulin Resistance)

**Figure 1:**
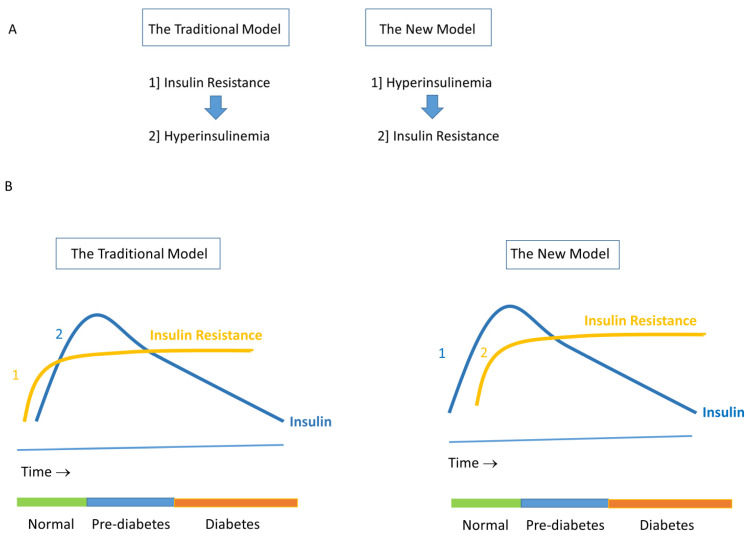
(**A**) In the traditional model, it was thought that insulin resistance preceded and led to hyperinsulinemia. In this model, hyperinsulinemia followed insulin resistance. In the new model, hyperinsulinemia is the triggering event and precedes insulin resistance. (**B**) In the traditional model, insulin resistance already started before the development of impaired glucose tolerance and type 2 diabetes. With time, the β-cell progressively lost its function. As a result, insulin secretion decreased, and frank type 2 diabetes developed. In the new model, hyperinsulinemia precedes insulin resistance. Hyperinsulinemia-induced insulin resistance develops during the conversion from normoglycemia to prediabetes. When with time the β-cell progressively loses its function, frank type 2 diabetes develops. Reproduced from [6].

For many years, the prevailing view was that (obesity-related) insulin resistance was the primary etiological factor in the sequence of metabolic changes leading to hyperglycemia and eventually to type 2 diabetes [67]. In this concept, hyperinsulinemia was viewed as secondary compensation by pancreatic β-cells to overcome the antecedent insulin resistance [7,8]. However, this view has been called into question in the past few years. Despite extensive research, no (plausible) mechanism can be identified to explain how insulin resistance could be responsible for increased insulin secretion in subjects with a normal glucose tolerance [68]. Therefore, a new hypothesis has been proposed, suggesting that chronic hyperinsulinemia not only precedes but also causes insulin resistance. In the second instance, this may lead to hyperglycemia and an increased fat mass and finally (in the long term) eventually to type 2 diabetes [6,67]. This scenario, known as the ‘insulin hypersecretion’ hypothesis, suggests that hyperinsulinemia is an important initiating event in the development of insulin resistance/type 2 diabetes [67] (Figure 1). In support of the insulin hypersecretion hypothesis, chronic hyperinsulinemia itself is capable of decreasing the number of insulin receptors on the cell surface, thereby reducing tissue sensitivity to insulin [69]. In addition, there is extensive evidence that chronic hyperinsulinemia (whether created by exogenous insulin infusion or by the stimulation of endogenous insulin) can lead to a specific defect in the insulin-mediated non-oxidative (glycogen synthetic) post-receptor pathway [35,70,71]. Overnutrition-induced hyperinsulinemia may be thus a driver of insulin resistance and type 2 diabetes [72]. In support of this latter scenario is the observation in mice that increased Akt-dependent insulin signaling was responsible for the development of insulin resistance after the start of a high-fat diet [73]. However, the development of insulin resistance in these mice could completely be reversed by administering a phosphatidylinositol 3-kinase inhibitor, suggesting that the blockade of insulin-mediated signaling may prevent insulin resistance [72,73] (see further below). Insulin resistance is in the ‘insulin hypersecretion’ hypothesis still a key component of numerous major diseases. In addition, it has been further suggested that hyperinsulinemia-induced insulin resistance can be considered an adaptive response of the body to maintain normoglycemia and prevent hypoglycemia in the presence of high insulin levels. It may function as a defense mechanism of the body by protecting critical tissues against insulin-mediated fuel overload, metabolic stress and overnutrition-induced injury [70,74,75].

## 7. Other Factors Contributing to Insulin Resistance

Subjects with a sedentary lifestyle are more prone to insulin resistance than physically active subjects [76]. Insulin action in the muscle and liver can be modified by acute bouts of exercise and by regular physical activity [77]. Trained subjects and those with high levels of physical activity exhibit high levels of insulin sensitivity/insulin action [77]. Regular physical exercise is an effective therapeutic strategy for improving insulin sensitivity by multiple mechanisms: it has the potential to increase cellular sensitivity to insulin and to reduce inflammation and obesity [78]. Excess adipose tissue/obesity results in an increased release of various ‘adipocytokines’ like leptin, resistin, tumor necrosis factor-alpha (TNF-α) and interleukin-6 (IL-6) [79,80]. Although an understanding of the precise role of distinct adipocytokines is still incomplete, an increased release of adipocytokines, by either adipocytes or adipose tissue-infiltrated macrophages, may lead to a chronic inflammatory state and in this way contribute to insulin resistance [79]. The most common cause of ectopic lipid accumulation in skeletal muscle and the liver is a chronic energy intake that exceeds chronic energy expenditure: as a result, there will be a spillover of energy storage from subcutaneous adipose tissue into the liver and the skeletal muscle (ectopic lipid accumulation) [81]. Recent studies using magnetic resonance spectroscopy have implicated ectopic lipid accumulation in the pathogenesis of insulin resistance in the muscle and liver [82]. Insulin resistance in muscle and liver cells appears to be a direct result of the intracellular lipid-induced inhibition of insulin-stimulated insulin receptor substrate (IRS)-1 tyrosine phosphorylation [81,82]. This then causes a reduced IRS-1-associated phosphatidyl inositol 3 kinase activity and thereby decreases insulin-stimulated glucose transport (Glut 4) activity (a hallmark of insulin resistance) [81,82]. It has been hypothesized that insulin resistance is the result of the accumulation of intracellular lipid metabolites (e.g., diacylglycerol and ceramides) in skeletal muscle and hepatocytes [81,82]. This hypothesis is supported by findings in mouse models of lipodystrophy and patients with congenital generalized lipodystrophy [83,84]. Patients with congenital lipodystrophy have a paucity of subcutaneous fat, severe insulin resistance and hypertriglyceridemia but a fatty infiltration of the liver and other tissues and a deficiency of adipocytokines [84]. Further evidence that ectopic lipid accumulation in the muscle and liver plays a causal role in the pathogenesis of insulin resistance is provided by studies showing that a reduction in ectopic fat is associated with the reversal of insulin resistance in these organs [85]. Moreover, the improvement in hepatic insulin sensitivity observed in patients with type 2 diabetes following weight loss is also accompanied by a significant reduction in intrahepatic fat without any changes in circulating adipocytokines (interleukin-6, resistin, leptin) [81]. All these observations suggest that the accumulation of intracellular lipid metabolites (e.g., diacylglycerol or ceramides) triggers insulin resistance and that neither obesity per se nor circulating adipocytokines are the main drivers of insulin resistance [81].

## 8. Hepatic Insulin Clearance and Hyperinsulinemia

Plasma insulin levels are determined by pancreatic insulin secretion and (hepatic) insulin clearance. Pulsatile insulin secretion regulates hepatic insulin clearance and, in so doing, systemic (peripheral) insulin delivery [86]. The liver preferentially extracts insulin delivered in pulses [86,87]. However, when the pulsatile pattern of pancreatic insulin in the portal circulation is diminished or almost disappeared, as can be found in prediabetes and early diabetes, systemic (post-hepatic) insulin levels will increase [86]. Normally, 20–80% insulin secreted by the pancreas into the portal vein is cleared during its first passage through the liver and never enters the systemic circulation [88]. Receptor-mediated insulin uptake followed by insulin degradation by hepatocytes is the basic mechanism of hepatic insulin clearance [89]. Unprocessed insulin passes the liver and is delivered into the systemic (post-hepatic) circulation to exert its actions in peripheral tissues (such as muscles and fat cells) [89]. Considerable intersubject variability in hepatic insulin clearance exists among individuals, and ethnic differences have been identified [88]. For example, hepatic insulin clearance is 67% lower in African Americans than European Americans [90]. Hepatic insulin clearance is a highly heritable trait in genome-wide linkage analysis, and several chromosomal loci have been identified that harbor genes regulating insulin clearance [91]. Carcinoembryonic antigen-related cell adhesion molecule-1 (CEACAM1) and insulin-degrading enzyme (IDE) are two key players in promoting hepatic insulin clearance [92]. Reduced hepatic CECAM1 level/function and/or reduced IDE level/activity impairs hepatic insulin clearance [92]. In a European/Portuguese population-based cohort, some IDE SNPs were strongly associated with postprandial hepatic insulin clearance in normoglycemic men [93]. In addition, it has been suggested that IDE polymorphisms governing postprandial hepatic insulin clearance can be affected by epigenetic modifications induced by hypercaloric diets, and this may contribute to an impaired capacity to fine-tune postprandial insulin levels [93]. Moreover, hepatic insulin clearance is a major and immediate regulator of systemic insulin concentrations responding within days to altered energy and carbohydrate intake [94]. For example, when a person switches to a high-caloric diet (particularly containing carbohydrates), hepatic insulin clearance can decrease within days, and this will increase peripheral insulin levels [94]. In addition, it has been reported that the fat accumulation of the liver is associated with impaired insulin clearance [95]. Reduced hepatic insulin clearance has been identified as a critical factor in the pathogenesis of hyperinsulinemia in the metabolic syndrome [96,97]. Recently, Bergman et al. hypothesized that inherited lower/reduced hepatic insulin clearance (rather than the overproduction of insulin by pancreatic islets) is the primary cause of peripheral hyperinsulinemia in at-risk individuals [90]. Bergman et al. further hypothesized that peripheral hyperinsulinemia exacerbates peripheral insulin resistance, and this causes extra stress for the pancreatic β-cells, which in susceptible individuals may eventually result in β-cell failure and the onset of type 2 diabetes [90].

## 9. Obesity and Type 2 Diabetes

**Figure 2:**
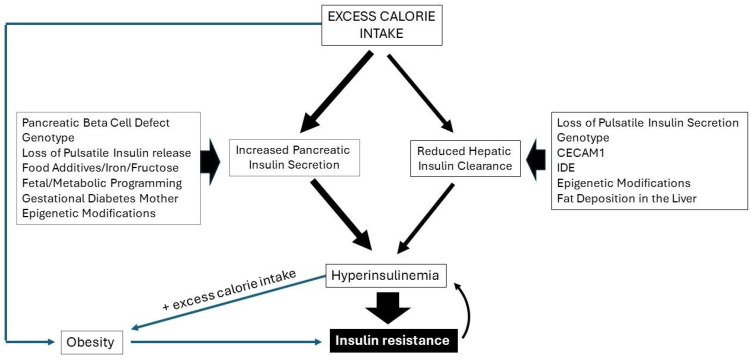
Factors involved in hyperinsulinemia and insulin resistance. Excess calorie intake may induce increased pancreatic insulin and/or a reduced hepatic insulin clearance. Hyperinsulinemia may be responsible for the development of insulin resistance and obesity. Obesity is also a consequence of excessive calorie intake. Obesity may further deteriorate insulin resistance, and this may further increase hyperinsulinemia. Pancreatic beta cell defects, certain genotypes, the loss of pulsatile insulin secretion, food additives, fructose, iron, gestational diabetes from the mother, epigenetic modifications and fat accumulation in the pancreas may play a pathogenetic role in increased pancreatic insulin secretion. The loss of pulsatile insulin secretion, certain genotypes (in particular CECAM1 and IDE), epigenetic modifications and fat accumulation in the liver may play a pathogenetic role in reduced hepatic insulin clearance.

Many adult subjects within Western populations show a gradual and progressive increase in weight with age and develop overweight/obesity during their lifetime due to a higher calorie intake than expenditure. Chronic overnutrition can lead to obesity [98]. For many years, the prevailing view has been that type 2 diabetes is a disease caused by obesity: obesity-induced insulin resistance was considered to be an important etiological factor in the development of hyperinsulinemia, hyperglycemia and eventually type 2 diabetes [67]. However, although both conditions often cluster, a potential (causal) relationship between obesity and type 2 diabetes is not very obvious [99]. People in countries such as Iceland, Mongolia and Micronesia develop obesity without diabetes, whereas people in countries such as India, Pakistan and China develop diabetes without obesity [100]. While obesity prevalence and diabetes prevalence often correlate, they are not concordant [12]. In addition, at present, most very heavy subjects in the Western world do not have type 2 diabetes, and only half of all subjects developing type 2 diabetes have a BMI in the obese range. In the United Kingdom Prospective Diabetes Study (UKPDS), conducted in the previous century, approximately one in three people (36%) newly diagnosed with type 2 diabetes had a weight in the normal range (i.e., a body mass index (BMI) of less than 25 kg/m^2^), whereas only 10% were obese [99]. This suggests that at least before 1991, there was no important role of obesity in the incidence of type 2 diabetes [99]. Furthermore, it has been found that subjects with type 2 diabetes, either nonobese or obese, show comparable reduced insulin-stimulated glucose utilization when compared with BMI-matched normoglycemic subjects, which is in support for no or only a minor pathogenic role of obesity in initiating insulin resistance and the incidence of type 2 diabetes [101]. On the other hand, hyperinsulinemia per se (often found in prediabetes and early stages of type 2 diabetes mellitus) causes weight gain/obesity by promoting lipogenesis, suppressing lipolysis and increasing lipid storage in adipocytes [102,103]. A causal role of hyperinsulinemia in obesity is further supported by the observation that a pharmacological reduction in insulin secretion lowers body weight in people who are obese [102]. Figure 2 summarizes several potential factors involved in hyperinsulinemia and insulin resistance. Excess calorie intake may induce increased pancreatic insulin secretion and/or reduced hepatic insulin clearance. Both increased pancreatic insulin secretion as well as reduced hepatic insulin clearance have been suggested to be etiological factors in the development of hyperinsulinemia and insulin resistance (see also legends in Figure 2 and previous paragraphs for more details).

## 10. Changes in Glucose Metabolism, Serum ALT and Triglyceride Levels before Developing Type 2 Diabetes

In the Whitehall II study, a prospective cohort study, repeated blood tests were collected from 6538 (71% male and 91% white) British civil servants without diabetes mellitus at baseline [104]. During a median follow-up period of 9.7 years, 505 subjects developed frank type 2 diabetes. Since blood samples had been taken and stored every year, it was possible to retrospectively assess the trajectories of the fasting glucose of all participants [104]. The mean blood fasting glucose gradually increased over a decade from 5.5 to 5.8 mmol/L but remained within the normal range [104]. In the 18 months preceding the diagnosis of diabetes mellitus, the mean fasting blood glucose level showed a relatively rapid and sharp rise and was 7.4 mmol/L at the time of diagnosing type 2 diabetes [104]. In the West of Scotland Coronary Prevention Study (WOSCPS), metabolic changes in parameters (glucose, lipids, liver enzymes, blood pressure and weight) potentially associated with the conversion to diabetes type 2 were retrospectively examined [105]. In all participants, serial glucose and other metabolic measures were collected every 6 months [105]. The 86 men, who converted to new-onset diabetes during the follow-up of the WOSCPS, were compared to 860 “nonconverters” matched for age and treatment. The mean (SD) increase in fasting glucose over 18 months in converters was 1.80 (1.52) mmol/L, compared with 0.10 (0.57) in nonconverters [105]. From all other parameters measured, only alanine aminotransferase (ALT) and triglyceride significantly increased in the 18 months prior to the onset of diabetes in converters compared to nonconverters [105]. Increasing ALT and triglyceride in the prediabetes stage may point to increased liver fat [106] (see further next paragraph). The authors therefore concluded that hepatic fat accumulation is probably the triggering event for the conversion to diabetes [105]. In support of this latter possibility, it has been found that elevated ALT is an early risk factor for developing type 2 diabetes, independent of obesity, body fat distribution, plasma glucose, lipid, AST, bilirubin concentrations and family history [107].

## 11. The Personal Fat Threshold Hypothesis and the Development of Type 2 Diabetes

As discussed, insulin is a major regulator of the partitioning of surplus calories by stimulating lipogenesis, suppressing lipolysis and increasing lipid storage in adipocytes. In the modern world, chronic positive caloric balance promotes hyperinsulinemia. The combination of excess calories (metabolic fuels) and chronic hyperinsulinemia initially shifts excess calories consumed by an individual towards deposition in adipocytes, preferentially in (safe) subcutaneous fat stores [108]. In response to overnutrition, the subcutaneous fat stores expand so that the excess calories are safely stored in adipocytes, which are specifically designed to store triglycerides, and this prevents toxic lipid accumulation in non-adipose tissues [109]. The ability to expand subcutaneous fat stores during development and upon overnutrition differs significantly between individuals and is gender-specific [110,111]. It has been hypothesized that there is an individual’s body fat set point. As a result, the storage capacity of the subcutaneous fat tissue may be limited: when due to overnutrition/excess calorie intake, the maximal subcutaneous adipose tissue expansion potential of an individual is reached, a further caloric surplus can no longer be stored in the subcutaneous fat stores and excess free fatty acids overflow to ectopic sites [112,113]. From that moment on, a caloric surplus is redirected toward other organs, leading to ectopic fat deposition: i.e., the storage of triglycerides in tissues other than the subcutaneous fat stores. Thus, excessive fat is stored in organs (such as the liver, pancreas, muscles and heart), which under healthy conditions, contain only small amounts of fat [112]. The observation that the metabolic control of type 2 diabetes in subjects with any BMI between 27 and 45 kg/m^2^ could become normal after losing a similar amount of weight led Professor Roy Taylor to propose the personal fat threshold (PFT) hypothesis [99]. He suggested that each individual has a PFT above which a person may develop type 2 diabetes [99]. As above discussed, the maximal storage capacity of subcutaneous fat stores varies significantly between individuals. Thus, different individuals have different PFTs [114]. The individual PFT determines the susceptibility of an individual to develop type 2 diabetes [99]. When an individual of any BMI can no longer store excess calories as subcutaneous fat, the PFT is crossed. This will initially promote ectopic fat deposition in the liver and induce an increased hepatic production of VLDL triglycerides. The exposure of pancreatic β cells to excess VLDL triglycerides results in an excessive accumulation of fat in the pancreas [99]. At a certain amount of (ectopic) fat accumulation in the liver and pancreas (and partly due to the negative effects of fat on hepatic insulin sensitivity/actions, pancreatic insulin secretion and muscle insulin sensitivity), prediabetes/type 2 diabetes will develop in susceptible individuals [99]. Importantly, the PFT per se is not directly related to BMI. Thus, the PFT hypothesis helps to explain why type 2 diabetes can also be found in subjects with a normal BMI [99] (see further below). In addition, it has been suggested (but at the moment not documented by studies) that different ethnicities have different PFTs, and this may help to explain why the prevalence of diabetes substantially differs among ethnic groups [115,116].

## 12. The Twin Cycle Hypothesis and the Etiology of Type 2 Diabetes

Humans also have a limited capacity to store (excess) energy as carbohydrates [117]. As a result, if the carbohydrate intake exceeds the oxidation and storage capacities of the body, surplus carbohydrates are converted in the liver to fat by de novo lipogenesis (DNL) [23,117]. The extent of portal hyperinsulinemia determines how much glucose is converted into fatty acid in the liver and will be used for DNL [23]. By converting excess carbohydrates and excess gluconeogenic substrates to triglycerides in the liver, DNL plays an important role in controlling glucose production and blood glucose concentration [117]. A carbohydrate-induced rise in hepatic DNL was found to be more pronounced when more than 50% of the carbohydrates in the diet were consumed as simple sugars [118]. Newly synthesized triglycerides in the liver can be either oxidized to deliver energy, exported as very-low-density lipoproteins (VLDLs) into the plasma or stored as triglycerides in the liver [23]. After excessive carbohydrate intake, the mitochondrial oxidation of free fatty acids is inhibited by high concentrations of malonyl-CoA produced during lipogenesis. As a result, newly synthesized triglycerides in the liver will be preferentially directed towards 1] storage, which will increase hepatic fat content, or 2] exported into the blood, which will increase plasma VLDL concentrations and ectopic fat deposition in other organs [23,119]. As DNL in the liver is directly stimulated by insulin, subjects with hyperinsulinemia will accumulate liver fat more readily than others and so contribute to the development of metabolic (dysfunction)-associated fatty liver disease (MAFLD) (formerly called nonalcoholic fatty liver disease (NASH)) [66,120]. Over many years, a modest increase in the fasting plasma glucose level chronically stimulates increased basal insulin secretion rates to maintain euglycemia, and this will further increase the conversion of excess calorie intake into liver fat [23]. This latter process is accelerated when a subject has also developed hyperinsulinemia-induced peripheral insulin resistance [23,121]. An increased liver fat content will cause relative (hepatic) insulin resistance, and this results in higher fasting glucose levels by decreasing the insulin-mediated suppression of hepatic glucose production. The mediators of hepatic insulin resistance are the toxic lipid intermediaries such as diacylglycerol and ceramides rather than stored triglyceride itself [122,123]. A fatty liver may also stimulate the overproduction and export of VLDL particles into the blood leading to the characteristic dyslipidemia associated with type 2 diabetes [121]. As discussed above, elevated VLDL levels increase fatty acid delivery to the pancreatic β cells. Excess fatty acid availability initially causes the pancreatic β cells to lose their ability to (acutely) increase insulin secretion in response to ingested food [23]. The progressive accumulation of fat in the pancreatic β cells may in the long term lead to the development of clinically manifest diabetes in susceptible individuals [23]. In conclusion, the twin cycle hypothesis postulates that chronic calorie excess initially leads to hyperinsulinemia and the accumulation of liver fat and in the second instance, to the spillover of fat into the pancreas [23]. These self-reinforcing cycles between the liver and pancreas may finally cause a reduction in pancreatic insulin secretion after meals and the onset of hyperglycemia in susceptible individuals, suggesting that type 2 diabetes is (mainly) caused by excess fat inside the liver and the pancreas [23].

## 13. Bariatric Surgery Can Induce Durable Type 2 Diabetes Remission

The UKPDS suggested that hyperglycemia progressively increased irrespective of the antidiabetic therapy used in the years following diagnosing type 2 diabetes [124]. Due to the progressive loss of β-cell function with disease duration, type 2 diabetes was considered a progressive disease [124]. However, an increasing number of studies have demonstrated that malabsorption-based techniques (RYGB gastric bypass and biliopancreatic diversion, both of which exclude the duodenum and jejunum from the alimentary circuit), but not restrictive techniques, can improve or induce the remission of type 2 diabetes within days of surgery [125,126,127]. Fascinating is that this can be found before significant weight loss has occurred [125,126,127]. The observed discrepancy in diabetes remission rates between RYGB gastric bypass and sleeve gastrectomy suggests a substantial role of the proximal intestine in type 2 diabetes remission and sustenance [128]. In further support of this latter possibility, a Billroth II (BII) surgical bypass after subtotal gastrectomy (which bypasses only a 15–20 cm segment of the duodenum) for cancer or intractable ulcers can also induce the remission of type 2 diabetes mellitus in patients without obesity and independent of weight loss [129]. Bariatric surgery has been shown to promote the restoration of physiological insulin secretion within weeks and to ameliorate insulin resistance during long-term follow-up [125,130]. Walter Pories was the first who reported that gastric bypass surgery normalized glucose metabolism/diabetes within one week after bariatric surgery, and this improvement in metabolism was observed long before there was any significant reduction in the mass of adipose tissue [131]. In addition, the remission of type 2 diabetes was durable, even though most of the “cured” patients remained obese [131]. However, the remission of type 2 diabetes was less likely to occur in patients who were older and who already had longer than 4 yr. type 2 diabetes [131]. Pories hypothesized that type 2 diabetes mellitus is due to an overstimulation of the pancreas by excessive gut signals supporting an important causal role of dietary factors in the development of hyperinsulinemia [132]. Pories concluded that a reduction in caloric intake (negative energy balance) was the main reason for the quick blood glucose control and remission of type 2 diabetes after gastric bypass [131]. Whether the exclusion of food from a large part of the small intestine as well as the presentation of undigested food to the mid-jejunum or other factors played additional roles in the improvement in endocrine pancreas and glucose tolerance was and is still unclear [131]. Pories concluded that if his hypothesis was true, increased insulin resistance in type 2 diabetes was not the cause but rather an effect of hyperinsulinemia [131]. This supports the above discussed concept that hyperinsulinemia precedes insulin resistance. In the Swedish Obesity Subjects study, bariatric surgery (predominantly gastric bypass and sleeve gastrectomy) brought the majority (72%) of the 343 subjects with type 2 diabetes at baseline into remission (i.e., fasting blood glucose < 110 mg/dL and no antidiabetic medications) [133]. However, after 15 years, the remission rate decreased to 30% [133]. In addition, a short diabetes duration at baseline was associated with higher diabetes remission rates during follow-up [133]. A meta-analysis of 4070 subjects by Buchwald showed a 78.1% remission of type 2 diabetes after gastric bypass [134]. The remission of type 2 diabetes observed after bariatric surgery does not support the prevailing view that pancreatic β-cells are already affected in the early stages of type 2 diabetes [131]. When pancreatic β-cells are still normal in the early stages of type 2 diabetes, this could help to explain why pancreatic β-cells can recover after bariatric surgery [131]. Although physiologically improbable, it has been postulated by some that the beneficial glycemic effects observed after bariatric surgery are mediated by increased post-meal glucagon-like peptide-1 (GLP-1) secretion. However, others have shown that albeit that GLP-1 changes post-surgery are associated, these are not a cause of early glycemia changes [135,136]. Decreased fasting insulin concentrations after Roux-en-Y gastric bypass (GB) surgery and sleeve gastrectomy (SG) are well documented [137]. So fasting insulin levels decrease dramatically, while glucose tolerance improves within 1–2 weeks after bariatric surgery. As mentioned, this is found well before any substantial changes in body weight have occurred [137,138]. The PFT hypothesis, as discussed above, helps us to understand why early type 2 diabetes can be reversed and normal glucose tolerance restored by bariatric surgery in the absence of substantial weight loss [99]. Initial reductions in glycemia and plasma insulin concentrations seem to be driven (in great part) by the negative energy balance postoperatively which limits substrate supply [138]. Within days after bariatric surgery, hepatic fat content decreases, and hepatic insulin clearance is restored. This latter improvement occurs independent of a change in peripheral insulin receptor sensitivity [137]. The sudden and rapid negative energy balance induced by bariatric surgery releases the chronic inhibitory effects of excess fat on pancreatic β-cell insulin secretion. As a result, pancreatic insulin secretion normalizes [139].

## 14. Caloric Reduction Can Also Induce Durable Type 2 Diabetes Remission

Based on the above discussed post-bariatric surgery effects on diabetes, Lim et al. hypothesized that a sudden negative energy balance is a strong signal to the body that changes metabolism [139]. Therefore, they tested whether initiating a very-low-caloric diet (VLCD; 600 kcal/day) for 8 weeks in 11 subjects with type 2 diabetes could normalize the insulin sensitivity of the liver and restore the normal acute insulin response of the pancreas. The normalization of both pancreatic beta cell function and hepatic insulin sensitivity in type 2 diabetes was achieved by dietary energy restriction alone, despite that all oral antidiabetic agents or insulins had been stopped at baseline [139]. This remission of diabetes was simultaneously associated with a decreased storage of excessive fat in the liver and the pancreas, supporting that most abnormalities underlying type 2 diabetes can be reversed in a short time by reducing dietary energy intake [139]. Petersen et al. were the first who demonstrated in type 2 diabetes a return of the normal levels of liver fat after dietary-induced weight loss [140]. They showed that moderate body weight loss (−8 kg) normalized fasting hyperglycemia in patients with poorly controlled type 2 diabetes: by reducing intrahepatic fat (−81%) by a hypocaloric diet, hepatic insulin clearance was improved. and hepatic insulin resistance was reversed after an average of 7 weeks [140]. This normalized basal glucose production rates, independent of any changes in insulin-stimulated peripheral glucose metabolism [140]. This observation emphasizes the existence of a tight relationship between liver fat content, hepatic insulin clearance and hepatic insulin sensitivity. The sudden fall of liver fat content also caused a decline in the (high) hepatic production rate of VLDL triglyceride [103]. The decline rate of hepatic VLDL triglyceride was so sharp that its supply to other organs in the body returned to normal [141]. As a direct result, excessive (ectopic) fat levels inside the pancreas gradually decrease, along with all other ectopic fat depots, and this restores the normal pancreatic insulin response to eating after 6–8 weeks [114,141,142]. In the Counterbalance study (Counteracting Beta-cell failure by Long-term Action to Normalize Calorie intake), subjects with type 2 diabetes and BMI 27–45 kg/m^2^ were initially treated with a VLCD for 8 weeks [143]. This was followed by a stepped return to an isocaloric diet, while a structured, individualized program of weight maintenance was provided [143]. Weight fell (98.0 ± 2.6 to 83.8 ± 2.4 kg; mean ± SD) and remained stable over 6 months (84.7 ± 2.5 kg) [143]. It was demonstrated that in the 40% of study participants who responded to a VLCD diet during 8 weeks by achieving fasting plasma glucose < 7 mmol/L, the remission of type 2 diabetes lasted for at least 6 months after the return to the isocaloric diet [143]. Responders showed a shorter duration of diabetes, and HbA1c fell from 7.1 ± 0.3 to 5.8 ± 0.2% (mean ± SD). Responders also showed a return of the first-phase insulin response, and the structured, individualized program of weight maintenance successfully prevented weight gain [143]. The Counterbalance study established that during weight stability, all abnormalities of liver and pancreas function remained reversed in the responders after 6 months, when weight regain was avoided [143]. The Diabetes Remission Clinical Trial (DiRECT) trial assessed the remission of type 2 diabetes during a primary care-led weight-management program [144]. The intervention consisted of the withdrawal of antidiabetic and antihypertensive drugs, total diet replacement (825–853 kcal per day formula diet for 12–20 weeks), stepped food reintroduction (2–8 weeks) and subsequently structured support for weight loss maintenance [144]. By this intervention, sustained diabetes remission (defined as HbA1c less than 6.5% (48 mmol/mol)) was obtained in nearly half of those with type 2 diabetes of up to 6 years duration [144]. In addition, sustained remission was linked to the extent of sustained weight loss after 24 months, and a 64% remission of type 2 diabetes was achieved in participants, who had maintained at least a 10 kg weight loss [144] (Table 2). 

**Figure 3:**
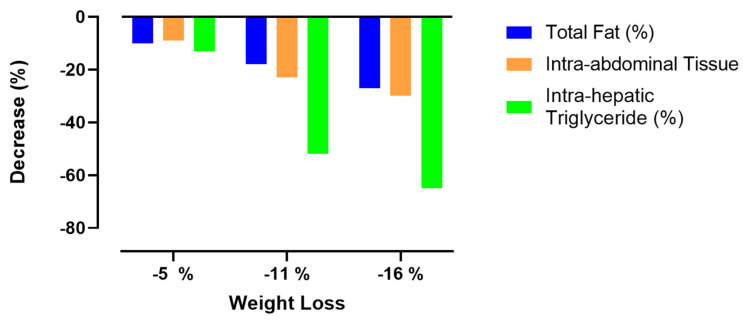
Effects of progressive weight loss (−5%, −11% and −16%) on fat content of total fat, intra-abdominal tissue and intrahepatic triglyceride (compared to baseline; (*n* = 9) *). After 16% weight loss, relative decrease in intrahepatic fat content was greater than relative decrease in intra-abdominal and total (subcutaneous) body fat content, whereas after 5% weight loss, there were no clear differences in loss of fat content between these three compartments. Fat content was measured as percentage via MRI. * Modified from [145].

**Figure 4:**
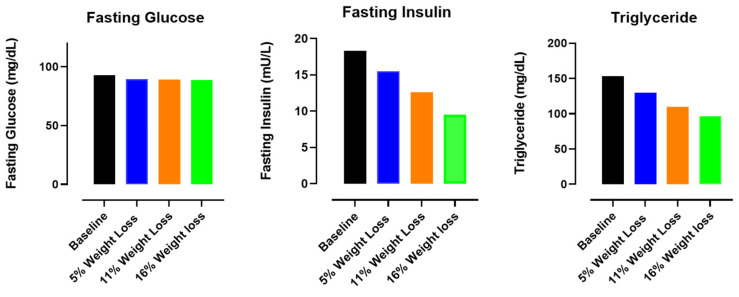
Effects of progressive weight loss (−5%, −11% and −16%) on fasting glucose, fasting insulin and triglyceride levels compared to baseline (*n* = 9) *. With progressive weight loss, fasting insulin and triglyceride levels significantly decreased, whereas fasting glucose did not change during increasing weight loss, suggesting improvement in hepatic insulin sensitivity. * Modified from [145].

It was further demonstrated that a weight loss of over 10–15 kg normalized ectopic fat within the liver and pancreas, thereby reversing the pathophysiological process underlying type 2 diabetes [141]. Although, a comparable weight loss and similar correction of intra-organ fat content was obtained in the whole intervention group (responders and non-responders), only the responders demonstrated early and sustained improvement in pancreatic β cell function [141]. Therefore, it was suggested that the lack of return to nondiabetic glucose control in non-responders was consistent with a more advanced, irreversible stage of pancreatic β cell dysfunction and disease duration in this latter group [141]. The ReTUNE study tested the personal fat threshold hypothesis in subjects with type 2 diabetes and a BMI < 27 kg/m^2^ [115]. The ReTUNE STUDY confirmed the personal fat hypothesis predictions also in subjects with a BMI < 27 kg/m^2^ by showing that 70% of the subjects with type 2 diabetes and with a normal or near-normal BMI could achieve the remission of type 2 diabetes by dietary weight loss despite stopping all glucose-lowering agents. Moreover, it was suggested that the pathophysiologic mechanisms underlying type 2 diabetes and its reversal were identical in people with a normal or raised BMI [115]. In this respect, it is important to realize that during diet-induced weight loss, fat is preferentially mobilized from intrahepatic and intra-abdominal fat stores, as opposed to subcutaneous fat tissue [142]. When the effects of 5%, 11% and 16.% weight loss were evaluated, it was found that weight losses of 5, 11 and 16% were associated with reductions in total fat mass of 10, 18 and 27%, respectively, reductions in intra-abdominal adipose tissue of 9, 23 and 30%, respectively, and reductions in intrahepatic triglyceride (measured as a percentage via MRI) of 13, 52 and 65%, respectively (see Figure 3 and Table 3 for more details) [145]. Intrahepatic triglyceride content became less than 5% after 11% weight loss (see Table 3; note when liver fat exceeds 5% of liver weight, in the absence of significant alcohol intake or other established risk factors for liver fat accumulation, there will be MAFLD [146]). Thus, intrahepatic fat stores lost relatively more fat after 16% weight loss than the total (subcutaneous) body fat compartment. However, the relative fat loss from the intrahepatic fat stores and the total (subcutaneous) body fat compartment did not differ after 5% weight loss. In addition, progressive weight loss caused dose-dependent alterations in fasting insulin, fasting triglycerides, insulin sensitivity and β-cell function [145] (see Figure 4 and Table 4). This study demonstrated that at least a 16% weight loss is sufficient to achieve type 2 diabetes remission but that it is not necessary to aim for a BMI in the normal range [145,147].

## 15. It Is Time for A Paradigm Shift in the Management of Type 2 Diabetes

The management of type 2 diabetes has historically focused on improving blood glucose concentrations and promoting exercise and weight loss, without specifically aiming to achieve the remission of type 2 diabetes. However, as above extensively discussed, type 2 diabetes should no longer be considered as a chronic, intractable, progressive disease. This paradigm shift is based upon pathophysiologic insights and substantiated by a series of clinical studies and observations, which have clarified important (new) mechanisms that cause type 2 diabetes: the personal fat threshold hypothesis suggests that type 2 diabetes develops when personal tolerance for fat levels in subcutaneous fat stores are exceeded [114]. Type 2 diabetes occurs as a result of the inability of the subcutaneous fat stores to further expand and accommodate excess fat following the chronic intake of excess calories [148]. When subcutaneous fat stores have become saturated, i.e., are unable to further accommodate to excess energy, the calories are stored as ectopic fat in the liver, pancreas and muscles, and frank type 2 diabetes will ensue in susceptible subjects [148]. There is strong evidence suggesting that type 2 diabetes can be put into remission by weight loss especially when this is started in the first years after diagnosing diabetes. At present, bariatric surgery is the most effective treatment for (selected) patients with type 2 diabetes to achieve glycemic control and type 2 diabetes remission (despite reduced or no glucose-lowering drugs) compared to intensive medical therapy and lifestyle interventions [149]. This translates into long-term benefits by reducing micro- and macrovascular complications and mortality [150,151,152]. Very-low-calorie meal replacement diets started early after diagnosis can also reverse the process that causes type 2 diabetes in a considerable number of subjects: a return of normal pancreatic β function prior to irreversible pancreatic β-cell changes can be achieved (within the first 6 years of diagnosis) by caloric (food energy) restriction to achieve a weight loss of around 15 kg [141]. This amount of overall weight loss seems necessary to reduce ectopic fat in the liver and pancreas in such quantities that it restores both normal pancreatic beta cell function and hepatic insulin sensitivity. Very-low-calorie diets should only be followed for a short period (2–3 months) and in the long term, be replaced by a healthy balanced diet that contains vegetables, fruit, whole grains, diary, seafood and nuts [153]. In fact, the changes in basal glucose production and basal glucose and insulin concentrations after very-low-calorie meal replacement diets are comparable to those reported 1 week after Roux-en-Y gastric bypass, which suggests that calorie restriction per se is sufficient to cause these changes. Roux-en-Y gastric bypass and very-low-calorie diets with formula meal replacement appear to be the most effective at helping people to limit their calories and lose more weight [153]. These two methods are more effective than other types of diets (Mediterranean diets, diets that replace only two meals a day with formula products, low-carbohydrate diets, vegetarian diets and low-glycemic index diets) and are currently the two methods that offer the best chance to achieve the remission of type 2 diabetes [153]. In addition, absolute weight loss, baseline pancreatic function and the duration of diabetes are the three main factors predicting the remission of type 2 diabetes [154,155]. Weight loss maintenance is notoriously difficult to consolidate by diet and exercise alone. Tirzepatide is a novel once-weekly glucose-dependent insulinotropic polypeptide/glucagon-like peptide-1 (GIP-1/GPL-1) receptor dual agonist, which recently has been approved as an adjunct to diet and exercise to improve glycemic control in type 2 diabetes [156,157]. Tirzepatide binds to both the GIP and GLP-1 receptors with different degrees of affinity [158]. This new agent may be a “game changer” in the treatment of type 2 diabetes [157,159]. In the SURPASS-1 clinical trial, type 2 diabetes patients, who were poorly controlled with diet and exercise alone, were treated with tirzepatide for 40 weeks [160]. Tirzepatide 15 mg caused a 2.07% HbA1c reduction and a 9.5 kg weight loss [160]. The proportion of patients who achieved HbA1c levels of ≤6.5% was 86% in the tirzepatide 15 mg group [160]. After 40 weeks of tirzepatide treatment, 31–52% (depending on the dose) of the participants with recent diabetes had returned to normoglycemia (HbA1c < 5.7%) compared to only 1% in the placebo group [160]. Moreover, in SURPASS-3, tirzepatide induced significant reductions in liver fat, visceral fat and subcutaneous fat as assessed by magnetic resonance imaging [161]. It should be underlined that at present, available data about tirzepatide are limited. More evidence is needed before this drug can be considered in subjects with newly diagnosed type 2 diabetes. In addition, there needs to be more clarity about tirzepatide’s cost-effectiveness, the duration of treatment needed to consolidate diabetic remission and the (long-term) risk/benefit balance of treatment with this drug. The primary prevention of type 2 diabetes (i.e., before it starts) is probably the most effective and cost-effective approach to halt the type 2 diabetes pandemic. When excessive fat within the liver and pancreas is indeed proven to be the main underlying mechanism driving type 2 diabetes, the development of type 2 diabetes can be avoided by preventing ectopic fat accumulation in the body. The prevention/reduction of ectopic fat accumulation in the liver and pancreas in an early stage may both prevent and cure type 2 diabetes. Therefore, future research should focus on the best strategies (for individuals and at the population level) to prevent ectopic fat accumulation and thereby type 2 diabetes.

## 16. Concluding Remarks and Future Perspectives

Type 2 diabetes has attained the status of a global pandemic in the past 40 years. In the same period, the daily energy intake has increased, and the quality of food has significantly changed. Chronic overnutrition and changes in the quality of food may have increased insulin secretion, decreased insulin pulses and reduced hepatic insulin clearance, thereby causing chronic hyperinsulinemia. The combination of chronic hyperinsulinemia and calorie excess may have induced insulin resistance and contributed to excessive ectopic fat accumulation in the liver and pancreas. In susceptible individuals, this may have led to hepatic insulin receptor resistance, the loss of pancreatic insulin secretion, hyperglycemia and the development of frank type 2 diabetes. Thus, the accumulation of excessive fat within the liver and pancreas seems to be a main underlying mechanism driving type 2 diabetes. Increasing evidence suggests that the prevention/reduction of ectopic fat accumulation in the liver and pancreas by interventions, like hypocaloric diets and/or bariatric surgery, may have disease-modifying effects in an early stage and thereby can induce a durable normoglycemic state. Therefore, preventing/reducing ectopic fat accumulation in the liver and pancreas in an early state may become a main treatment goal in the management of type 2 diabetes. 

## Figures and Tables

**Table 1:** Hyperinsulinemia at baseline is a significant predictor of prediabetes/type 2 diabetes at follow-up.

Type of Study	Number of Subjects Included	Mean Follow-Up (Years)	Outcome	Reference
Prospective	5042	3	type 2 diabetes	[14]
Prospective	309	8	type 2 diabetes	[15]
Prospective	5176	7.2	type 2 diabetes	[16]
Prospective	319	6.4	type 2 diabetes	[17]
Prospective	266	6	type 2 diabetes	[18]
Prospective	647	12–15	type 2 diabetes	[19]
Prospective	515	24	prediabetes/type 2 diabetes	[21]

**Table 2:** Relationship between weight loss and remission of type 2 diabetes in direct study.

Weight Loss	Participants Achieving Remission * of Type 2 Diabetes at 12 Months	Participants Achieving Remission * of Type 2 Diabetes at 24 Months
<5 kg	3.6%	5.2%
5–10 kg	33.9%	28.8%
10–15 kg	57.1%	60.0%
>15 kg	86.1%	70.0%

Progressive weight loss increased remission percentage of type 2 diabetes. * Remission of diabetes was defined as HbA1C less than 6.5% (<48 mmol/mol) after withdrawal of antidiabetic drugs at baseline (independent of status at 12 months). Modified from [144].

**Table 3:** Effects of increasing weight loss (−5%, −11% and −16%) on body composition (fat content measured as percentage via MRI) and fasting glucose, fasting insulin and triglyceride levels compared to baseline (*n* = 9) *.

	Weight (kg)	BMI (kg/m^2^)	Body Fat (%)	Intrahepatic Triglyceride (%)	Fasting Glucose (mg/dL)	Fasting Insulin (mU/L)	Triglyceride (mg/dL)
Baseline	103.8	37.7	48.3	8.5	92.7	18.3	153
**5%** Weight loss	97.9	35.5	46.3	7.4	89.4	15.5	130
**11%** Weight loss	92.5	33.6	44.2	4.1	89.3	12.6	110
**16%** Weight loss	87.0	31.6	42.2	3.0	88.6	9.5	97
*p*-value	<0.001	<0.001	<0.001	<0.001	0.288	<0.001	0.003

From these results, it can be concluded that intrahepatic fat percentage decreased relatively more than total (subcutaneous) body fat with increasing weight loss. Intrahepatic fat percentage became less than 5% when weight loss was more than 10% (left panel). In addition, increasing weight loss causes dose-dependent significant decrease in fasting insulin and triglyceride levels, whereas fasting glucose did not change, suggesting improvement in insulin sensitivity (right panel). Results are shown as means for normally distributed variables or medians (for not normally distributed variables). Note: when liver fat exceeds 5% of liver weight, there is metabolic (dysfunction)-associated fatty liver disease (MAFLD). * Modified from [145].

**Table 4:** Effect of increasing weight loss (−5%, −11% and −16%) on multi-organ insulin sensitivity and β-cell function compared to baseline (*n* = 9) *.

	Insulin AUC (mU/L·min)	Insulin Clearance Rate (pools/min)	Β-Cell Function	Palmitate Ra Suppression (%)	Glucose Ra Suppression (%)	Glucose Rd Stimulation (%)
Baseline	12,365	0.36	6860	53	71	168
**5%** Weight loss	12,950	0.40	8130	49	77	207
**11%** Weight loss	11,137	0.41	10,607	52	76	326
**16%** Weight loss	9534	0.48	11,107	58	80	311
*p*-value	0.024	0.016	0.003	0.009	0.026	0.002

With increasing weight loss, insulin AUC, insulin clearance rate and β-cell function significantly increased (left panel). With increasing weight loss, adipose tissue insulin sensitivity, liver insulin sensitivity and skeletal muscle insulin sensitivity significantly improved (right panel). Results are shown as means for normally distributed variables or medians (for not normally distributed variables). Measures of organ-specific insulin action were assessed by using a two-stage hyperinsulinemic–euglycemic clamp procedure in conjunction with infusion of stable isotopically labeled tracers. Index of β-cell function was calculated as product of Φ-total assessed during oral glucose tolerance test and relative increase in glucose Rd during high-dose insulin infusion (stage 2) of clamp procedure. AUC = area under curve; palmitate Ra suppression = insulin-mediated suppression of palmitate rate of appearance [Ra] in plasma (measure of adipose tissue insulin sensitivity); glucose Ra suppression = insulin-mediated suppression of glucose rate of appearance in plasma (measure of liver insulin sensitivity); glucose Rd stimulation = insulin-mediated stimulation of glucose rate of disappearance [Rd] from plasma (measure of skeletal muscle insulin sensitivity). * Modified from [145].

## Data Availability

Not applicable.
